# Emission Properties of Fluorescent Nanoparticles Determined by Their Optical Environment

**DOI:** 10.3390/nano5020895

**Published:** 2015-05-29

**Authors:** Kelvin Chung, Snjezana Tomljenovic-Hanic

**Affiliations:** School of Physics, The University of Melbourne, Parkville, Victoria 3010, Australia; E-Mail: kelvinc@student.unimelb.edu.au

**Keywords:** fluorescence, nanoparticles, emission rate

## Abstract

The emission rate of a radiating dipole within a nanoparticle is crucially dependent on its surrounding refractive index environment. In this manuscript, we present numerical results on how the emission rates are affected for nanoparticles in a homogenous and substrate environment. These results are general, applicable to any refractive index distribution and emitter.

## 1. Introduction

In the last few years, study into fluorescent nanoparticles (NPs) has become a distinctive subject of research. This increase in interest is driven by many applications of these materials in industry, medicine, information technology, energy storage, sensing, biomedicine, and many others [1,2]. Numerous studies, both experimental and theoretical, have been undertaken to explore the effect of local environment on spontaneous emission properties of emitters and their corresponding lifetimes. The role of a high refractive index substrate in close proximity to the dipole has been well established in references [3,4,5].

The lifetime of an excited state, or the spontaneous emission rate for the transition considered, is changed when an atomic system is placed close to a metal surface or a dielectric interface. This has been demonstrated in experiments within reference [6] and analytical expressions have been derived for isotropic media interfaces [3]. The effect of encapsulation of a dipole inside a NP has been extensively explored theoretically [7,8]. If the size of the NP is much smaller than the emission wavelength this effect can be easily calculated analytically based on the Mie theory for the emission rate of a dipole placed at a given location in a dielectric sphere of an arbitrary size [7]. Numerical studies on the modification of the spontaneous emission rate as a function of the size and shape of the medium as well as the position of the emitter in it were also reported [8].

**Figure 1:**
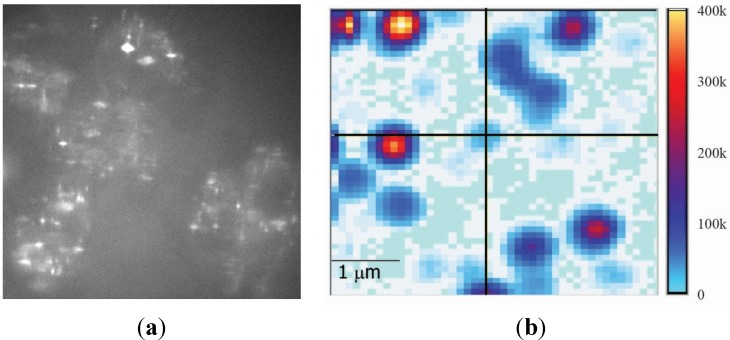
Confocal scans of fluorescent nanoparticles (NPs): (**a**) Zinc oxide NPs in skin cells and (**b**) Nanodiamonds on a silicon substrate [13]. Reproduced with permission from [13], Copyright 2014, Biomedical Optics Express. These are examples of two main configurations considered in this manuscript: free standing nanoparticles in medium (liquid) and NPs deposited on the high refractive index substrate.

Figure 1 shows representative confocal scans of two main configurations that will be considered in this manuscript. These represent experimental conditions where it is difficult to distinguish the role of a single parameter since there are a multitude of factors that contribute to the measurement. For example, if the NP is deposited on a substrate, the emission rate depends on many parameters including the refractive index (both real and imaginary) of the substrate, the upper space refractive index, and the refractive index of encapsulating material. Additionally, collection efficiency depends how and from where the emission rate is measured. In many cases, the emission rates are compared for largely different scenarios. For instance, the emission rate of nanodiamond (ND) has been compared when deposited on different substrates, most commonly silicon or glass, and coated with different materials [5,9,10,11]. Only recently, studies involving aerogel suspension, with the refractive index close to air, simulated a ND without any influence of substrate or coating/supporting material [12]. Measured emission rates and lifetimes are substantially different to previously reported studies, which did not take into account entirely surrounding optical environment [12].

In this manuscript, we theoretically investigate influence of optical environment to emission properties of NPs. The scenarios considered represent an intuitive flow of logic in which pertinent information is collated. Our findings are general and they are applicable to any fluorescent nanoparticle. In Section 2.1, we describe our model and method. In Section 2.2, we investigate the effect of encapsulation of a dipole in a high refractive index NP, as well as the double encapsulation effect. In Section 2.3, the effect of absorption, both of environment and nanoparticle, is investigated. In Section 2.4, the role of high refractive index substrate is summarised in addition to the change of the refractive index of the top layer.

## 2. Results and Discussion

### 2.1. Model and Method

**Figure 2:**
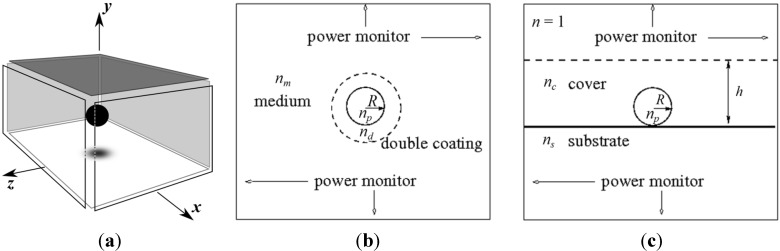
(**a**) The three-dimensional diagram of the numerical simulation, where the black coloured sphere represents the NP at the centre enclosed by a “cube” of power monitors. Each face of the cube is offset from original position to emphasise that a NP is at the centre. The two main configurations considered for the manuscript showing the 2D refractive index distribution of (**b**) NP in homogenous medium, and (**c**) NP on a substrate. These are cross-sectional slices of each respective refractive distribution which is enclosed by the power monitor cube.

Two main configurations are considered in this manuscript as illustrated in Figure 2. First, a NP in medium with the refractive index *n_m_* is free-standing, see Figure 2b. An optional double encapsulating layer is denoted by the dashed line and has the refractive index *n_d_*. The second configuration considered is a NP located on the surface of a substrate with the refractive index *n_s_*, see Figure 2c. The upper medium is assumed to be air, with the refractive index *n* = 1. An optional cover film, denoted by the dashed line in Figure 2c, has the refractive index *n_c_* and thickness *h*. All media are assumed to be homogeneous and isotropic. The NP radius is *R* = 25 nm unless stated otherwise, and the refractive index is *n_p_*.

The theoretical model is based on dipole encapsulated within a spherical NP [7] and is treated as an oscillating electric dipole in the proximity of a plane interface [3,4]. The configuration shown in Figure 2b does not contain a plane interface and is described by Reference [7]. The radiated power from a dipole within a NP will be different in a homogenous (*i.e.*, free space or air) environment compared to a refractive index distribution with a planar interface. For both configurations, the dipole was assumed to be in the centre of the NP.

Pertaining to Figure 2c’s configuration, the dipole will radiate into upper and lower regions with respect to the substrate. The following inequality holds for the refractive index distribution for this configuration: *n* (air region) < *n_c_* < *n_s_*. Evanescent waves exist since the relative refractive index, *n_rel_* ≡ *n_s_*/*n_m_* > 1, and are reflected from the substrate interface. These reflected waves transform into plane waves upon immediate reflection into the film cover [14]. The substrate effect also needs to be coupled to the encapsulation of the electric dipole by the NP and the total radiated power is dependent on the refractive index ratio of *n_p_*/*n_c_* [7].

As mentioned above, the power radiated by a NP is calculated by a model of a classical oscillating electric dipole encapsulated in a NP, located in the centre of the NP. The dipole’s orientation, when deposited on a substrate, is described by the angle between its axis and the interface. Here we consider parallel and orthogonal polarisations. Three-dimensional Finite-Difference Time-Domain (FDTD) on a cluster of machines is used for the emission rate calculations [15]. The dipole is a point-like object, which is modelled by a current source within the FDTD simulation domain. There is a rectangular box consisting of power monitors, which is surrounding a NP. The computational domain size is 500 nm × 500 nm × 500 nm with a perfectly-matched layer width of 1 μm that acts to absorb any radiation incident upon and not reflect back into the computational domain. For the numerical simulations, satisfactory convergence is obtained by using 20 points per NP size.

We would also like to acknowledge that Förster resonance energy transfer (FRET) could also play a role, especially when the dipole and substrate are in close proximity, in the emission rate properties. If the substrate’s fluorescence wavelength, after some arbitrary excitation, from optically active defects does not overlap with the dipole’s absorption properties, then the contribution from FRET is insignificant. Additionally, the phonon pathway for the nanoparticles was not considered and therefore no vibrational or rotational states were involved. The aforementioned mechanisms were not considered in the numerical simulations.

### 2.2. Dipole Encapsulation

**Figure 3:**
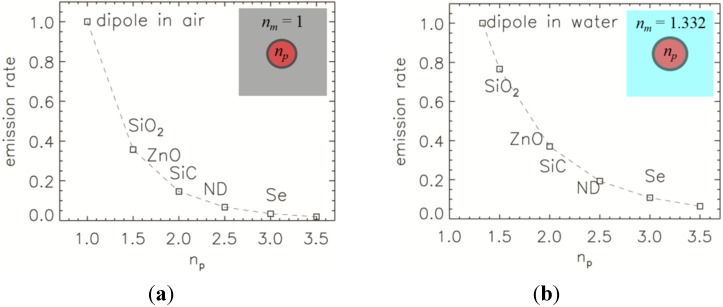
Emission rate, normalised to the emission rate of the dipole in (**a**) air and (**b**) water without any encapsulation, as a function of the refractive index of NP, *n_p_*. Both figures contain an inset of the schematic of the refractive index distributions considered in the simulations. The materials along the dashed curve silica (SiO_2_), zinc oxide (ZnO), silicon carbide (SiC), nanodiamond (ND), and selenium (Se).

As discussed in introduction, decades ago it was pointed out that the emission rate of a dipole in air and an encapsulated dipole is significantly different. In Figure 3, numerical results for the emission rate, normalised to the emission rate of a non-encapsulated dipole, as a function of the refractive index of encapsulated material is presented. The size of NP is 50 nm in diameter and the refractive index range spans from *n_p_* = 1, e.g., dipole in air, to *n_p_* = 3.5. Some of materials are denoted along the curve, from SiO_2_ NP with *n_p_* = 1.45 to Se NPs with *n_p_* = 3.0. The emission wavelength is arbitrary and taken to be λ = 532 nm. It is evident from Figure 3b that the larger the refractive index of encapsulated material the larger is the reduction in dipole emission. It drops from 100% for a dipole in air to 1.9% for a dipole encapsulated in material with the refractive index *n_p_* = 3.5 compared to dipole emission in air.

In the first instance, we considered NPs in air only, to avoid inclusion of any other effect, such as a substrate or fluid. Since NPs cannot be suspended in air and probed, it must be within some material with an air-like refractive index. This was achieved by Inam *et al*. [12] where NDs were embedded into silica aerogel (*n =* 1.05). Their work showed that the mean lifetime increased when the ND was within the aerogel compared to when it was on a coverslip.

Additionally, Rogobete *et al.* [8] theoretically studied how the spontaneous emission rate is affected depending on the size, shape and the position of the dipole within a NP. They assumed the NP had a refractive index of *n* = 1.59 (λ = 628 nm). At *n* = 1.59, our results show that the normalised emission rate is just below 0.4 and this matches Rogobete *et al.*’s results for a dipole placed at the centre of spherical NP of the same size.

Next, we consider NPs suspended in water with a refractive index of *n_m_* = 1.332 (at λ = 532 nm), that is close to the refractive index of any culture media used in biomedical experiments. Figure 3b shows how a water environment has a similar effect to an air environment but the reduction is smaller as the refractive index difference between the environment and encapsulating material is smaller. In this case, the emission rate drops from 100% for the dipole in water to 6% when encapsulated with *n_p_* = 3.5.

**Figure 4:**
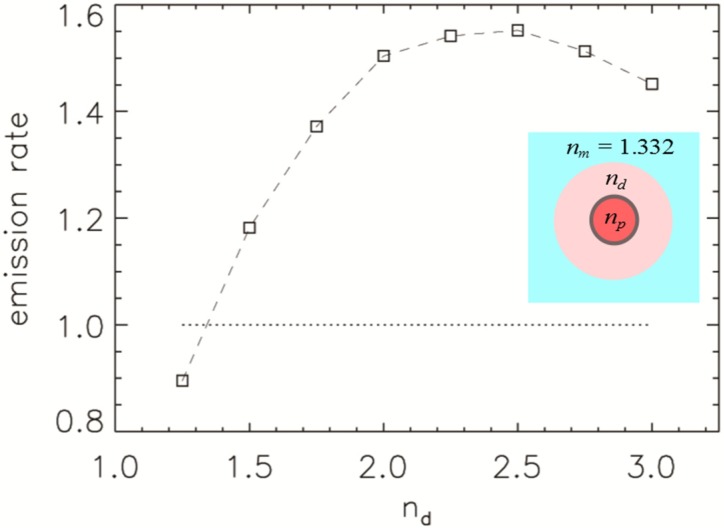
Emission rate of NPs with the refractive index *n_p_* = 2.5, and radius *R* = 25 nm (normalised to the emission rate of the particle without the second layer) in water as a function of the second layer refractive index, *n_d_*, and a total radius *R* = 50 nm. Region above the dotted line indicates enhanced emission. The inset shows a schematic of the double encapsulation refractive index distribution.

We now investigate double encapsulation where the second layer commonly has some other purpose than a change of optical environment. Double encapsulation has been used for the suppression of blinking, such as for smaller NDs [16] and metal-oxide NPs [17]. In other applications, quantum dot bioactivity can be improved with an additional coating [18]. In Figure 4, we show the emission rate, normalised to the emission rate of the NP without a second layer, as a function of the second layer refractive index. NPs are considered to be in water and the refractive index of the first layer is *n_p_* = 2.5 and radius *R* = 25 nm.

Any encapsulation with the second layer refractive index larger than the refractive index of environment, here *n_m_* = 1.332, enhances the emission rate, region above the dotted line in Figure 4. It increases from 1.00 at *n* = 1.332 to 1.55 at *n* = 2.5, which is the refractive index of the first layer. Therefore, the largest enhancement is achieved by increasing the size of NP rather than encapsulating the particle. However, it is common that double encapsulation is done to suppress fluorescence intermittency (“blinking”) as mentioned earlier. Another example of experimental work by Chen *et al*. [19] has shown that the fluorescence intermittency can be suppressed for CdSe quantum dots if they’re encapsulated with monolayers of inorganic material resulting in core-shell “giant” quantum dot.

There has also been recent work done by Bray *et al*. [20], where they encapsulated ND with phenol-ionic complex (*n* ≈ 1.9 [21]) and observed enhanced photoluminescence, *i.e.*, decrease in lifetime, from the nitrogen-vacancy centre defect within ND. Any coating of the ND changes the local refractive index that the light encounters and, therefore, affects the dipole emission rate as well.

### 2.3. Absorption

**Figure 5:**
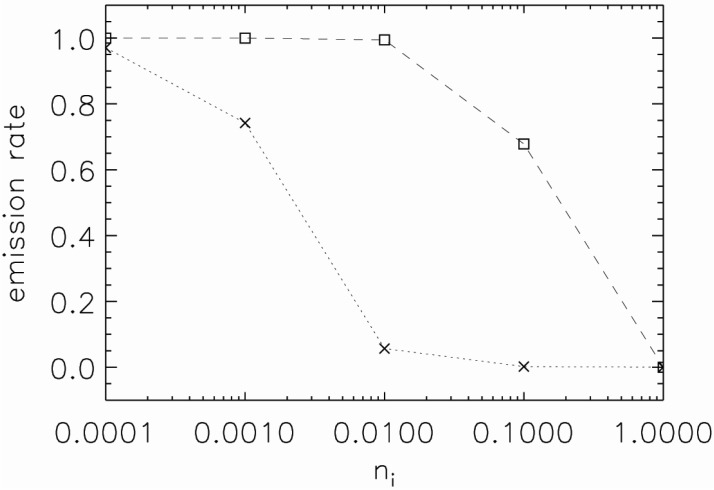
Emission rate of NP with *n_p_* = 3.0 (normalised to the emission rate of the dipole in water with *n_i_* = 0) as a function of the imaginary refractive index of NP (the dashed line) or the imaginary refractive index of environment (the dotted line).

In reality, both a NP and environment can be absorbing. This may significantly affect emission properties of the dipole. To quantify this effect we include an imaginary part *n_i_* to the refractive index of NP or environment. The real part of the refractive index of NP is fixed at *n_p_* = 3.0 and environment is water, *n_m_* = 1.332. Changes in emission rate with induced absorption are plotted in Figure 5 as a function of the imaginary refractive index.

Initially, the NP emission rate is less affected by induced absorption in the NP itself, up to *n_i_* = 0.01 there is almost no effect on emission. However, for an absorptive environment there is significant drop in emission rate at *n_i_* > 0.001. The emission rate drops to 6.7% compared to the emission rate of non-absorbing environment at *n_i_* = 0.01. The role that absorption plays is further discussed in the next section.

### 2.4. Substrate Role

Solid NPs are most commonly deposited on a cover slip (*n_s_* = 1.54) or a silicon surface (*n* = 3.875, *n_i_* = 0.01 at λ = 637 nm). If the dipole is in close proximity to the high reflective surface the emission rate is significantly enhanced. The effect depends on the distance from the surface, the real and imaginary refractive index of the surface. Here, we consider only changes in the emission rate. The radiation pattern and collection efficiency has been investigated in detail, see references [4,22].

**Figure 6:**
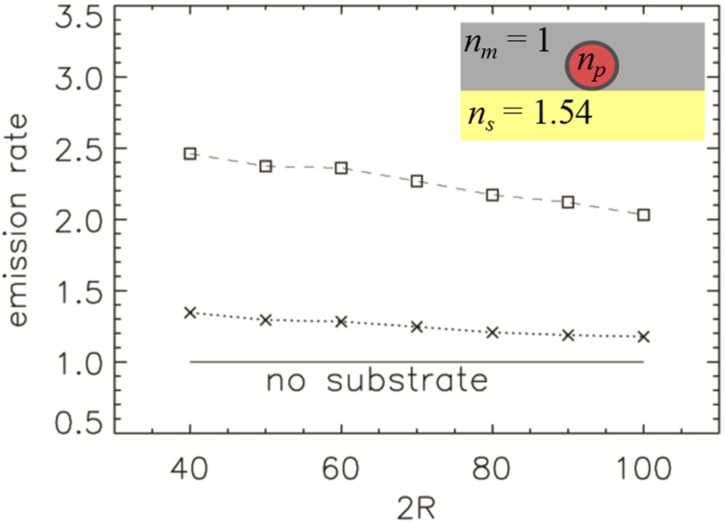
Emission rate of nanodiamond (ND) with a refractive index of *n_p_* = 2.42 (normalised to the emission rate in air without substrate) for the orthogonal (squares) and parallel polarisation (crosses) as a function of the NPs size (dipole is in the centre of ND). The ND is on top of a cover slip with a refractive index of *n_s_* = 1.54, *i.e.*, Figure 2c configuration. The inset shows a schematic of the refractive index distribution.

In Figure 6, we plot the emission rate, relative to the emission rate without the substrate, of a NP with *n_p_* = 2.42 deposited on the cover slip with the refractive index *n_s_* = 1.54. The size of the NP varies from 2*R* = 40 nm to 2*R* = 100 nm. The smaller the particle size, the closer the dipole is to the reflecting surface. Therefore, the enhancement is more pronounced for smaller particles. The orthogonal polarisation is more enhanced than the parallel one.

**Figure 7:**
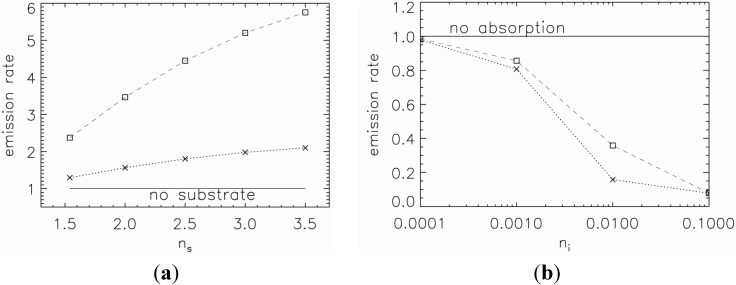
Emission rate of 50 nm large ND (*n* = 2.42) on a substrate for orthogonal (squares) and parallel (crosses) polarisation as a function of (**a**) real refractive index *n_s_* (*n_i_* = 0) and (**b**) imaginary refractive index *n_i_* with fixed real refractive index at *n_s_* = 3.875.

The refractive index difference between the substrate and upper media (see Figure 2c) determines how much light is reflected from the high refractive index substrate. The larger the refractive index difference, more light will be reflected. This effect is shown in Figure 7a where the emission rate, relative to the emission rate without a substrate, is plotted as a function of the real refractive index of the substrate. The effect is more pronounced for the orthogonal polarisation of the dipole, reaching an almost six-times increase for the refractive index *n_s_* = 3.5.

The imaginary refractive index also plays a role, in particular, in determining how much light is absorbed by the substrate. In Figure 7b, we plot the emission rate, relative to the emission rate without absorption, as a function of substrate’s imaginary refractive index. The real part of the refractive index is fixed at *n_s_* = 3.875. For both polarisations, the emission rate significantly reduces with an increase of the imaginary refractive index. At *n_i_* = 0.01, the emission rate for the parallel polarisation is less than 20% and for the orthogonal polarisation is 35% compared to the non-absorbing substrate.

**Figure 8:**
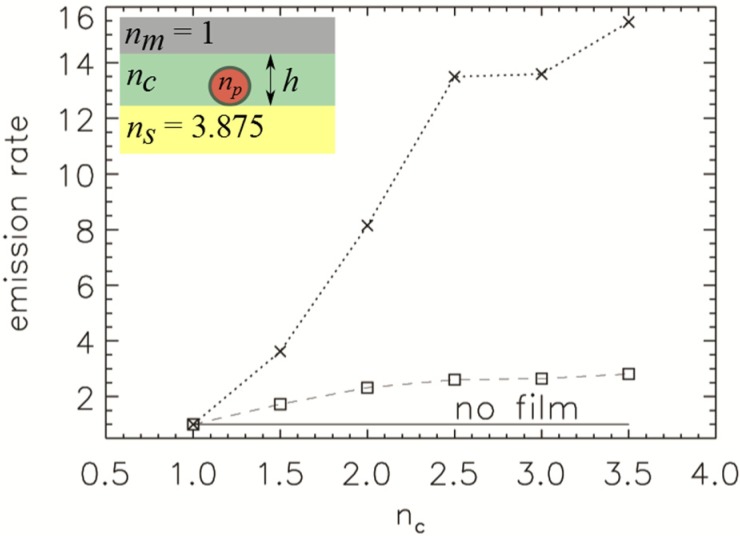
Emission rate of 50 nm large NP on a substrate with *h =* 200 nm thick cover film for orthogonal (squares) and parallel (crosses) polarisation as a function of cover film refractive index *n_c_*. The refractive index of non-absorbing substrate is *n_s_* = 3.875. The inset shows a schematic of the refractive index distribution.

The last configuration we consider in this manuscript is an optional film cover with a refractive index *n_c_* > 1 which is illustrated in Figure 2c with the dashed line. The thickness of the film is chosen to be *h* = 200 nm which is thick enough to cover the NPs. We investigate the emission rate of a NP with a refractive index *n_p_* = 2.42 (diamond), relative to the emission rate without the film, for both polarisations as a function of the cover film refractive index, see Figure 8. The emission rate of the orthogonal polarisation changes from 1.0 for *n_c_* = 1 to 2.1 for *n_c_* = 3.5. The emission rate of parallel polarised dipole is more significantly affected, increasing from 1.0 at *n_c_* = 1, to the 15.8 enhanced emission rate at *n_c_* = 3.5.

The coated film decreases the refractive index contrast between the upper and lower regions surrounding the NP and, therefore, reflectivity from the silicon substrate is reduced. The enhancement solely comes from decreasing the refractive index difference between the NP and surrounding environment. This behaviour agrees with previous work conducted by Khalid *et al*. [13] where NDs were coated with a thin film of silk (*n_c_* = 1.54) on top of a silicon substrate. Their results showed an increase in the emission rate of the dipole within the ND which agrees with our numerical results.

## 3. Conclusions

It has been shown through numerical simulations that changing the refractive index distribution that encapsulates the NP can modify the emission properties of NPs. Although the physics of emitters has been extensively studied in the past, there exists no systematic collection of the scenarios considered in this manuscript. These results are general and widely applicable to any arbitrary refractive index distribution that encapsulates a NP.

For a “free-standing” NP, the emission rate tends to decrease with an increasing refractive index environment. If a NP is double encapsulated, there is a maximum emission rate achieved when the second layer matches the refractive index of the NP that effectively increases the size of the NP. When absorption is included, the emission rate of the NP is subject to larger changes when an imaginary refractive index is included in the surrounding environment compared to within the NP.

The substrate that the NP is deposited was also found to modify the emission rate. The refractive index properties of the substrate also affect the emission rate where a real and imaginary refractive indexshowing an overall trend of increasing and decreasing emission respectively. If a thin film covers the NP there was an increase in the emission rate with increasing refractive index of the film.
